# Preparing newborn screening for the future: a collaborative stakeholder engagement exploring challenges and opportunities to modernizing the newborn screening system

**DOI:** 10.1186/s12887-021-03035-x

**Published:** 2022-02-12

**Authors:** Sara M. Andrews, Katherine Ackerman Porter, Donald B. Bailey, Holly L. Peay

**Affiliations:** RTI International, 3040 E Cornwallis Rd. Research, Triangle Park, NC 27709 USA

**Keywords:** Newborn screening, Stakeholder engagement, Public health policy, Transformative therapies

## Abstract

**Background and objectives:**

Projections that 60 transformative cell and gene therapies could be approved by the U.S. Food and Drug Administration (FDA) within 10 years underscore an urgent need to modernize the newborn screening (NBS) system. This study convened expert stakeholders to assess challenges to the NBS system and propose solutions for its modernization.

**Methods:**

NBS stakeholders (researchers, clinicians, state NBS leaders, advocates, industry professionals, and current/former advisory committee members) participated in one of five mixed-stakeholder panel discussions. Prior to panels, participants completed a survey in which they reviewed and ranked NBS challenges generated from relevant literature. During panels, participants deliberated on challenges and explored potential solutions. Pre-panel survey data were analyzed descriptively. Data from panel discussions were analyzed using a rapid qualitative analysis.

**Results:**

Median scores of the ranked challenges (1 = most important) reveal the top three most important barriers to address: critical missing data for NBS decision-making (Median = 2), burden on state NBS laboratories (Median = 3), and the amount of time required for state-level implementation of screening for new conditions (Median = 4). Panel discussions were rooted in recurring themes: the infant’s well-being should be the focal point; the transformative therapy pipeline, although undeniably positive for individuals with rare diseases, is a threat to NBS capacity; decisions about modernizing NBS should be evidence-based; additional financial support is required but not sufficient for modernization; and modernization will require participation of multiple NBS stakeholders. This final overarching theme is reported in depth, including expertise, coordination, and collaboration challenges facing NBS and novel approaches to oversight, partnership, and coordination that were suggested by participants.

**Conclusions:**

This study engaged representatives from multiple stakeholder groups to generate potential solutions to challenges facing NBS in the United States. These solutions provide a rich starting point for policy makers and other stakeholders who desire to maximize the impact of new transformative therapies for babies, families, and society.

**Supplementary Information:**

The online version contains supplementary material available at 10.1186/s12887-021-03035-x.

## Background

Public health decision-making is inevitably complicated because of the involvement of multiple stakeholders, such as federal, state, and local government, researchers, health care providers, advocates, and the public at large. In the United States, history has demonstrated the challenges of unifying and mobilizing stakeholders when a public health system needs to adapt to external forces such as advances in technology or the onset of new infectious diseases [1, 2]. Newborn Screening (NBS) presents a classic example of a complex public health system needing to adapt to best serve its intended beneficiaries: babies and families. Over the past 50 years, NBS has saved or improved the lives of countless babies by identifying rare, but serious medical conditions presymptomatically and referring them for immediate treatment. However, the system in its current state is unprepared to adapt to an approaching opportunity and challenge: the advent of a growing number of transformative therapies targeting rare conditions. The number of conditions that are likely to be candidates for NBS once they have an associated therapy threatens to overwhelm the system unless stakeholder partnerships are leveraged and processes are changed.

NBS in the United States requires coordination between state and federal governments, which increases the complexity of implementation and change. The federal government has oversight over the national Recommended Uniform Screening Panel (RUSP), and the process of adding a condition to the RUSP involves contributions of both government and nongovernment stakeholders. Conditions are recommended for the RUSP by the Advisory Committee on Heritable Disorders in Newborns and Children (ACHDNC), a committee consisting of 15 voting members appointed by the Secretary of Health and Human services (10 individuals with relevant expertise and 5 individuals who represent federal agencies that fund or support aspects of NBS, such as Centers for Disease Control and Prevention [CDC]) and additional representatives from organizations that intersect with infant and child health (e.g., American Academy of Pediatrics). At the state level, legislatures, departments of public health, NBS laboratories, and advisory committees are key players in decisions about adding RUSP conditions to each state’s screening panel, and screening and follow-up procedures for each condition vary by state.

Other NBS stakeholders include rare disease advocates and policymakers, who worked together to enact the federal legislation that made NBS a public health program and who continue to advocate to fund and enhance the program on federal and state levels. Families and advocacy groups also play an important role in advocating for conditions to be added to the RUSP and state NBS panels. Additionally, prior to implementing NBS for new conditions, there is a need for researchers to collect natural history data, academic and industry sponsors to develop and test treatments, and private companies and public institutions to develop diagnostic tests and screening protocols. Finally, health care providers have the critical role of performing diagnostic testing, communicating with families about their child’s condition, connecting babies to treatment, and providing long-term follow-up services.

Despite successful collaboration of these stakeholders to build and sustain NBS, the system now faces a disruptive, albeit positive, force: the transformative therapy pipeline for rare conditions. At least 60 cell and gene therapies are projected to be approved by the Food and Drug Administration (FDA) by 2030, and FDA recently reported more than 1000 Investigational New Drugs (INDs) applications for cell and gene therapy treatments are currently on file [3, 4]. NBS could ensure timely access to these treatments. However, the need for federal approval and state implementation of screening for so many conditions would almost certainly overwhelm the current system. Collecting sufficient evidence to meet the criteria for RUSP approval [5] can take years. And once a condition is added to the RUSP, there is wide variability in how long it takes each state to approve a condition for its panel and allocate funding for implementation. For example, X-linked adrenoleukodystrophy was nominated to be considered for the RUSP in 2012 and added to the RUSP in 2016 [6], but currently only 23 states screen for the condition [7]. Thus, it can take years to achieve nationwide screening for even one condition. The degree to which this problem could be exacerbated by the transformative therapy pipeline is sobering and underscores the critical need to engage all NBS stakeholders in modernizing the system.

In this study we convened a series of multistakeholder panels to engage NBS experts and advocates in collectively prioritizing challenges to NBS modernization and proposing solutions to enable realizing the potential of transformative therapies within the NBS system.

## Methods

### Aims

We employed a multistakeholder expert panel approach to address three aims:

- **Aim 1.** Prioritize and explore the most impactful barriers to realizing the potential of transformative therapies within the NBS system.
- **Aim 2.** Generate potential solutions to barriers of NBS modernization.
- **Aim 3.** Rate the feasibility, acceptability, and sustainability of implementation of solutions identified across the panels.

The first two aims were addressed through multistakeholder panel discussions described herein. The third aim was addressed by a follow-up survey completed by panel participants, which is reported elsewhere [8].

### Study design

We modeled our approach after several methodologies that have implemented group discussion among heterogeneous stakeholders for brainstorming or problem-solving [9–13]. Focus groups, which are traditionally and intentionally composed of individuals with similar experiences, are an established method for eliciting stakeholder perspectives [14]. However, when the objective is systems change, discourse between stakeholders with diverse priorities and experiences can result in a more holistic understanding of the breadth of a problem and the generation of more viable solutions [15]. Accordingly, we selected mixed-stakeholder discourse as the preferred methodology to explore barriers and solutions to modernizing the NBS system. Participants in each of our panels represented five NBS stakeholder groups. During the panel sessions we did not aim to achieve thematic saturation or consensus, but instead to facilitate initial cross-stakeholder consideration of challenges and brainstorm solutions, with the expectation that ongoing engagement of a larger number of stakeholders would be needed to refine solutions.

Research activities were framed around this transformative therapy scenario: *It is 2030—ten years from today. Thirty or more new transformative gene or cell therapies have been approved by the FDA to treat monogenic, non-oncology rare disorders. Please consider the following assumptions. These assumptions may not reflect future reality but will be useful to frame our upcoming group discussion.*

- *Each treats a different genetic disorder.*
- *Each has a valid screening assay that is not prohibitively costly.*
- *The therapies are curative or significantly disease modifying if given early in life, but much less or not effective if given later.*
- *The longer-term risks and duration of efficacy are unknown.*
- *Assume that the cost of the therapies will be completely covered by payers (*e.g.*, insurance, Medicaid).*

### Recruitment

NBS experts (i.e., individuals highly experienced with some aspect of NBS) were nominated by a consortium of funders and RTI researchers and invited to participate. We aimed to recruit approximately 50 participants, representing 10 individuals from each of 5 stakeholder groups:

1. NBS researchers or clinicians
2. State NBS directors or program leaders
3. Representatives of patient advocacy organizations
4. Representatives of pharmaceutical or diagnostic companies
5. Current and former members of federal or state advisory committees

### Approach

Prior to initiating data collection, the investigators conducted a literature scan to identify and summarize challenges to NBS that are reflected in published literature; these are available as Additional File 1. Data collection was conducted between December 2020 and January 2021 and included three phases.

#### Pre-panel survey

An online survey included basic demographic questions and a rating activity where participants indicated their familiarity with and expertise in various aspects of NBS. Additionally, participants were asked to rank a list of NBS challenges stemming from the summarization of the published literature. Participants were asked to indicate which challenges were the most important to address to achieve NBS for 30 new disorders in 10 years, based on the context of the transformative therapy scenario (Additional File 1).

#### Multistakeholder expert panels

We then convened five mixed-stakeholder expert panels of 7–10 participants per panel. Each 90-min panel discussion was conducted virtually using Zoom, a web conferencing platform. Audio and video were recorded. Investigators used a semistructured interview guide to address the following:

- *Exploration of each panel’s prioritized challenges to NBS modernization.* Investigators displayed the results of the pre-panel ranking of challenges for each of the panels (i.e., each panel viewed their own panel members’ aggregated ranking results). The moderator asked participants to elaborate on the most highly prioritized challenges and to make a case for any challenges that were missing from the list.
- *Solutions*. Participants were guided to explore potential solutions to the most highly prioritized challenges and to consider the acceptability, feasibility, and sustainability of those solutions based their own expertise, priorities, and experience with NBS.

#### Post-panel survey

Approximately one month after each panel discussion, participants were asked to respond to a second online survey, reported in a separate article.

Participants were asked to draw on all of their NBS experiences and perspectives during their participation (i.e., not attempt to reflect the experiences or attitudes of one stakeholder group). Each participant was offered a $100 gift card for participation. Some participants declined the incentive. This study was determined to be exempt by the RTI International Institutional Review Board (IRB).

### Analysis

Pre-panel survey data were analyzed using descriptive statistics. Multistakeholder panel discussions were analyzed qualitatively by investigators who moderated and took notes during all five panels (HLP and SMA, respectively) and a third analyst (KAP). The investigators, all of whom have expertise in qualitative methods, conducted a rapid assessment process, which is a team-based qualitative inquiry that uses triangulation and iterative data analysis to quickly develop an understanding of the data [16]. Rapid qualitative analyses are increasingly used for health services and implementation research in which there is a need to quickly, but rigorously, synthesize findings for use in policy and practice decision-making [17–20]. Investigators implemented the rapid assessment using a matrix-based approach that included audio-recordings and detailed notes from each panel discussion [17, 21–23]. Specifically, recordings and notes were used to develop structured summaries of each panel. Preliminary coding of the structured summaries was completed using codes derived from the moderator guide. The third analyst reviewed transcripts to quality check the summaries and the preliminary coding, transferred summaries into a data matrix organized by the challenges and solutions proposed by each panel, and incorporated supplementary notes and quotes. Using a consensus process, the analysis team reviewed the matrix to refine the coding into meaningful categories and compare challenge and solution themes across panels [17, 24]. The study Principal Investigator (DB) reviewed the analysis and provided an additional expert perspective on the interpretation of the summary findings.

Here we describe primary themes that emerged; this report is not inclusive of all challenges and solutions that were discussed.

## Results

### Participants

Forty-two experts consented to participate. Participant demographics and self-identified stakeholder group are reported in Table 1. Stakeholders’ ages ranged from between 35 years and 75 years or older. Most stakeholders (71%) had a doctorate degree. All were from the United States or Canada.

**Table 1:** Participant Demographics

Expert Panel Characteristics (*n* = 42)	
	Count (%)
**Age (years)**	
35 to 44	8 (19)
45–54	12 (28)
55–64	10 (24)
65–74	7 (17)
75 or older	2 (5)
Prefer not to answer	3 (7)
**Gender**	
Female	23 (55)
Male	17 (40)
Prefer not to answer	3 (9)
**Education Level**	
Doctorate Degree	30 (71)
Master’s Degree	4 (10)
Bachelor’s Degree	8 (19)
**Race**	
White	36 (86)
Asian	3 (7)
Black or African American	1 (2)
Other	1 (2)
Prefer not to answer	1 (2)
**Ethnicity**	
Hispanic	2 (5)
Not Hispanic	40 (95)
Self-identified Stakeholder Group*	
Patient Advocacy	15 (36)
Research Leader	16 (38)
Advisory Committee	18 (43)
State Leader	16 (38)
Industry Leader	10 (24)

*Self-identified, sometimes more than one group; two participants did not self-identify and their roles were determined by research staff based on current job title

### Ranking of challenges in pre-panel survey

Aggregated results of the ranking activity, across all panels, are reported in Table 2. A median ranking score was calculated for each of the challenges, with lower score indicating higher importance of the challenge for addressing the transformative therapy scenario. Of the nine challenges, critical missing data for NBS decision-making was the highest ranked challenge (Median = 2, range 1–9), followed by burden on state NBS laboratories (Median = 3, range 1–8) and the amount of time required for state-level implementation of screening for new conditions (Median = 4, range 1–7).

**Table 2:** Ranking the most important challenge to address to modernize NBS (*n* = 41)*

Challenges identified in literature	Median ranking score (range) ^a^
Critical data will be missing	2 (1–9)
There will be implementation burden for state NBS laboratories	3 (1–8)
State-level implementation will take considerable time	4 (1–7)
There will be RUSP review burden	5 (1–9)
Yet-to-be determined disorder heterogeneity will complicate clinical decision making	5 (1–9)
Major expansion of state bioinformatics capabilities will be needed	6 (2–9)
Accessible state follow-up programs will need to be developed and implemented	6 (1–8)
There will be uncertainties about long-term benefits and risks of transformative therapies	7 (1–9)
Educational materials and resources will need to be developed	9 (1–9)

^a^ Instructions were to rank the challenges (from 1 to 9) in order of importance to allow rapid implementation of NBS for 30 conditions, where 1 = most important

* Missing data for one participant

### Overarching themes

Although all panel discussions were framed around the same hypothetical scenario, the content of discussions varied by nature of being prompted based on highly ranked challenges from the pre-panel survey, facilitated by a semi-structured guide, and largely driven by participants. Across all panel discussions, stakeholders expressed shared attitudes and beliefs around which they framed their discussion of NBS’s challenges and solutions. Overarching themes and exemplary quotes can be found in Table 3.

**Table 3:** Overarching Panel Themes: Exemplar Quotes

	Themes	Exemplar Quote
3.1.	The infant’s well-being should be the focal point for the NBS system as new solutions are developed and implemented.	“Because in the end, we’re trying to save a child. We’re trying to save a baby...Who are we, if we fund basic science, basic science moves to translational science, then moves to clinical trials, INDs, IRBs, clinical trials and approved therapies to the FDA. And we cannot figure out a way to deliver the therapy to a baby? But we have just spent a billion dollars to develop the therapy and answer the science that can bring a life-saving therapy to a baby, but we’re going to let the newborn screening be the hiccup? That doesn’t even make any sense to me.” (Panel 2, Participant 12)
3.2.	The transformative therapy pipeline is a threat to NBS system capacity, which already suffers from inefficiencies and delays because of burden on federal and state systems.	*a. The RUSP review process:* “What I always tell other groups [preparing RUSP nominations] when they come and ask me, is that if you have a projection day from when you have a therapy, you need to be working simultaneously on newborn screening several years before you think you’re going to have an approved drug, because there’s multiple different levels. You have to get prepared and get buy-in from a large community of different stakeholders before you’re ever going to have enough data and evidence and comfort level to have your condition put on the RUSP.” (Panel 2, Participant 11) *b. State-level factors affecting implementation:* One of the initial assumptions [in the scenario provided], was that there is a method for screening available, but that doesn’t take into account what has to be done at the state level. That method has to be scaled up, and all the procurement involved with that. And just because there’s a valid method doesn’t mean that a particular state lab can just kind of turn it on one day. So that’s part of the time commitment involved. (Panel 4, Participant 33)
3.3.	Decisions about how to modernize the NBS system should be evidence-based.	“If we’re looking towards a 2.0 system, what are the concepts around the 1.0 system that we need to retain? And I think the existence of a national advisory body with appropriate expertise is something that ought to be retained. I don’t think we want to go back to a circumstance where all the states are making their own decisions. Traditionally, we’re not always evidence-based.” (Panel 3, Participant 16)
3.4.	Additional financial support is required but is not sufficient for successful NBS modernization.	“We have to really be thinking that [NBS] is embedded in a US healthcare system, which is fairly disjointed and where, despite what we were told [in the transformative therapy scenario], money is very important, and access is quite variable…I just wonder what the next steps look like.” (Panel 3, Participant 23)
3.5.	Successful modernization will require the participation and coordination of multiple stakeholders and organizations in the development, implementation, and ongoing evaluation of new solutions.	“In order to move forward and to recreate newborn screening we need a group like this with all of these different perspectives coming together to hash out sort of what the issues are from the various viewpoints. I agree 100% with what [Advisory Committee] said, and with [Research Leader] and [State Leader]. Really, this whole personalized medicine versus newborn screening versus diagnostic testing is really at the crux of where the group is coming from, from their various perspectives.” (Panel 3, Participant 24)

#### The infant’s well-being should be the focal point for the NBS system as new solutions are developed and implemented

Stakeholders reinforced their shared desire to connect babies to life-saving treatments and shared frustration at current and potential future barriers to this objective (Table 3, Quote 3.1.).

#### The transformative therapy pipeline is a threat to NBS system capacity, which already suffers from inefficiencies and delays because of burden on federal and state systems

Stakeholders acknowledged that the time-consuming nature of evidence review by the ACHDNC and implementation of new conditions by states is not scalable in the context of the transformative therapy pipeline (Table 3, Quotes 3.2.a. and 3.2.b.).

#### Decisions about how to modernize the NBS system should be evidence-based

Stakeholders valued evidence-based decision-making. They emphasized that critical data are missing in the current system and that these missing data further threaten the ability of NBS to adapt to anticipated therapeutic advances. Stakeholders thus underscored the importance of solutions that result in data generation using standardized approaches. They also endorsed ongoing assessment of the evidence generated by such approaches, and evidence-based refinements or revisions to the system (Table 3, Quote 3.3.).

#### Additional financial support is required but is not sufficient for successful NBS modernization

Stakeholders reinforced the vital need to provide sufficient financial support at all levels of the system: to allow for critical data to be collected and analyzed; to make it feasible for the ACHDNC to more rapidly review a larger number of conditions; for states to implement screening; and for states to support and improve their follow-up programs. But while financial resources are critically needed, the stakeholders agreed that an infusion of funding would not address NBS challenges unless implemented along with other changes to the system (Table 3, Quote 3.4.).

#### Successful modernization will require the participation and coordination of multiple stakeholders and organizations in the development, implementation, and ongoing evaluation of new solutions

Participant discussion revealed a shared understanding that NBS is a multifaceted system that requires engagement and collaboration among stakeholders with differing motivations and norms, including federal agencies, researchers, policymakers, treatment facilities, state NBS laboratory and follow-up teams, patient advocates, and the public. Successful NBS requires a broad range of expertise, and diverse stakeholders must be part of planning, implementation, and evaluation of new approaches to modernize NBS modernization (Table 3, Quote 3.5.).

This final overarching theme of collaboration, coordination and expertise-sharing was reflected in much of the panel deliberations, as described below. Exemplary quotes that address the theme of expertise and coordination challenges can be found in Table 4.

**Table 4:** Expertise and Coordination Challenges: Exemplar Quotes

	Theme	Exemplar Quote
4.1.	State/federal coordination challenges	“The role the federal government plays is to provide the resources for doing the studies that are necessary to develop best practices or other things that might help states have a better understanding or to build their follow-up program, for example…. I think the federal government plays an important role, but I think that unless the whole system changes, it’s a state-driven public health program and they have the opportunity to take what’s out there and meld to what’s the best for rural state or urban state or a whole bunch of different things that might change how they apply some of these things.” (Panel 4, Participant 30)
4.2.	Expertise-related implementation challenges	“But even adding new positions is a tremendous challenge, trying to find a qualified person to do newborn screening when the current position descriptions are really based on 20 years ago. And so, I think it’s positions, it’s resources—it’s expertise. We’re talking about a whole new—potentially new—paradigms of testing. The newborn screening labs are not very limber in terms of putting new stuff out. They do a great job with what they do, but they’re not very limber in terms of adding new things very rapidly.” (Panel 5, Participant 36)
4.3.	Public education and awareness challenges	“We call newborn screening this huge public health success, yet so few people know about it. And now that more people do know about it, I mean, we do have those fears of the public that come in [privacy concerns, fears about misuse of dried blood spots]…” (Panel 1, Participant 2)

### Expertise and coordination challenges

#### State/federal coordination challenges

As a national public health service, NBS requires leadership from the federal level and coordination across 50 states and territories. Stakeholders described the RUSP as an “unfunded mandate,” underscoring the challenge of federally initiated policies driving decision-making for state implementation, but without accompanying financial support or policies to standardize implementation and follow-up. For each condition added to the RUSP, individual state laboratories must expend time and resources on preparatory activities such as verifying screening methodologies and developing standard operating procedures (SOPs) for screening and follow up before implementing a screening approach (Table 4, quote 4.1.).

As a result, there can be wide variability in the rollout of screening for new conditions across states and the possibility that babies in neighboring states will be screened for a different set of conditions. Longitudinal data, particularly around child outcomes after follow-up, are often missing because of the variability in state data collection requirements and lack of coordination between the medical specialists who provide treatment and the state follow-up program. Stakeholders also described lack of federal guidance and coordination as a contributor to gaps in follow-up data and lack of standardized follow-up practices across states. Finally, stakeholders acknowledged that there is often a lack of coordination and communication between federal agencies that collect data and make decisions regarding the funding and implementation of NBS, such as FDA, CDC, and the Health Resources & Services Administration.

#### Expertise-related implementation challenges

A successful NBS program requires a broad range of expertise. Stakeholders described how screening, confirmatory testing, and follow-up have become more complex with the advent of new testing methodologies and technology advances (e.g., molecular techniques for confirmatory testing, genotype-specific treatments), causing a need for expanding expertise and a more specialized workforce (Table 4, Quote 4.2.). Additionally, as NBS expands, follow-up personnel and institutions that provide treatment and management require additional expertise in many rare conditions and across all organ systems.

Simultaneously, NBS has been impacted by staffing issues associated with high turnover rates at state laboratories, in some cases attributed to lower salaries as state employees compared to academic or industry laboratories. NBS is also one of many competing public health priorities that state laboratories are managing. Highly competent laboratory staff and follow-up personnel are necessary for the advent of 30 new conditions being added to the RUSP, and stakeholders doubted states’ ability to recruit and maintain the requisite staffing and expertise to implement so many conditions.

#### Public education and awareness challenges

NBS stakeholders also described challenges related to public awareness and education. Stakeholders acknowledged NBS as a highly impactful public health initiative but recognized that the impact and benefits of NBS are not widely known by the public or by state legislature*.* Furthermore, the public perception of NBS has been threatened by recent lawsuits and legislative battles over privacy and allowable uses of dried blood spots collected for NBS (Table 4, Quote 4.3.).

Stakeholders described a need to improve public knowledge of NBS and build appreciation for the benefits and impact of NBS. There was an emphasis on expanding conversations beyond the typical stakeholders and reaching those who have the greatest stake in NBS: families.

### Novel approaches to oversight, partnership, and collaboration

Stakeholders’ proposed solutions to these challenges led to discussions of intersecting topics, including expertise-sharing, capacity-building, and good communication, addressing a broader theme of leveraging stakeholder collaboration to modernize NBS. Additional concepts explored improved integration of federal, state, and nongovernment systems. Exemplary quotes addressing the theme of novel approaches to oversight, partnership, and collaboration can be found in Table 5.

**Table 5:** Novel Approaches to Oversight, Partnership, and Collaboration: Exemplar Quotes

	Theme	Representative Quote
5.1.	Expand collaborative pilot studies to test implementation of screening	“If you’re trying to bring 30 conditions on by 2030, how do you get there...you’re going to need to get to some populated areas. We need inclusivity. We need diversity…. We need to think about these bigger states that have diversity and diversity of cultures, and how do we capture the most babies that we can through studies as quickly as possible?” (Panel 2, Participant 12)
5.2.	Develop expertise-sharing models	*a.* *Create regional NBS laboratories with expertise and capacity in particular methodologies:* “Actually, just in the past couple of weeks I’ve talked to a few programs who are dying with their staffing…I think it is not only the number of staff, but the quality and competencies of staff, especially as we do look at our technology is becoming more complex, and the post-interpretation needs becoming more complex. So, I do think this whole ability for states to do their own screening is something we really have to think about and maybe start thinking about doing a more regionalized model that kind of disperses the need for these high [complexity] staff [roles].” (Panel 4, Participant 32) *b.* *Create or expand collaboration between state NBS programs and universities and academic centers:* “I really liked the idea of bringing in different centers, because I think that could be really advantageous, especially for maybe a state that doesn’t have geneticists. So the idea of being able to bring in different centers for the purpose of whatever that condition is, could be extremely advantageous.” (Panel 4, Participant 29)
5.3.	Develop a public-private partnership to increase resources and reduce burden on the NBS system	“When people say something like ‘public,’ ‘private,’ they think about…a pharmaceutical company or private lab working with a state lab. But I think what we’re really talking about is even wider. Stakeholders are thinking about different kinds of partners, whether those are academic medical centers, whether those are private health clinics, whether those are telehealth for genetic counseling programs. I think this idea of a multi-tiered stakeholder or multistakeholder collaborations are even beyond private, public, medical center, university...I do think that this idea of networked partnerships is going to be incredibly important, and it already is in both research and in the clinical world.” (Panel 4, Participant 30)
5.4.	Other innovative solutions: A “conditional RUSP”	“[A condition] might have a low threshold for initial approval, if you can develop a good rationale for why this ought to be on the roster, then go ahead and approve it, and then collect the data. After it’s been implemented, and people are doing more than a pilot study here, and more than say five states there, and re-review the data in a couple of years. This requires people to think about taking conditions off the RUSP, which is pretty much vanishingly rare now, but shouldn’t be. So how about a system in which you augment the development of the data by early approval, but then express a willingness to take it off.” (Panel 3, Participant 16)
5.5.	Improve education and public opinion about NBS	*a. Reframe rare diseases as a collective public health burden:* “Chronic diseases, such as diabetes or cardiovascular, [affect] 1 in 10 Americans, and the idea that the public health system is very set up to deal with diabetes because 1 in 10 people in America have diabetes. So, newborn screening, which has a role to play in identifying those with rare diseases [could] extrapolate the idea that 1 in 10 people have rare diseases. It’s possible…that the public health departments in the United States receive federal funding for something like a rare disease program, just like they currently have a diabetes program. And then from there you could enable newborn screening to be an element of the rare disease program, just like diabetes screening and different efforts exist. And so, if you have more awareness of how prevalent rare disease is then it could enable a lot more support in the states from the feds in terms of funding to do more of the things that we probably all dream of doing.” (Panel 5, Participant 39) *b. Create a NBS public relations campaign:* “And I’m not just saying that we’re needing to educate when we’re pregnant and expecting. I mean, the education start as young as elementary and high school and why this is taking place and that this is an absolute great thing that you’re being offered to help ensure the health and safety of an unborn child...And so, I think that we really need to go some at some of these things we need to go back to the basics and really figure out how we can improve the education over time to ensure that families are not opting out of this amazing program.” (Panel 1, Participant 5)

#### Expand collaborative pilot studies to test implementation of screening

Stakeholders proposed expanding pilot studies as a way to collect data or increase state laboratory capacity before adding new conditions to states’ panels, which may improve the feasibility of screening for multiple new conditions at once. One proposed model was to support several large or diverse states in conducting collaborative, multi-state pilot studies to obtain missing data on a new condition, or set of conditions, to support evidence-based decision-making prior to adding conditions to the RUSP. This model would leverage cross-state collaboration and information-sharing and likely involvement of federal and academic partners (Table 5, Quote 5.1.).

Another proposed model was for individual states to offer screening for conditions soon to be added to the RUSP, prior to adding them to the state’s screening panel, through a consented research study similar to existing pilot studies such as Early Check (North Carolina) or ScreenPlus (New York) [25, 26]. This model would particularly leverage collaboration between state and academic partners to provide access to screening for some babies prior to full state implementation. Collection of critical missing data would be an important objective, with an understanding that it would be more challenging to achieve representative participation for research that requires parental permission (i.e., not opt-out) for newborn participation.

Use of either of these pilot study models may allow for new academic/public health partnerships to emerge while generating critical data and providing opportunities for states to use research funding (e.g., from NIH) to expand their expertise, obtain necessary equipment, develop and evaluate screening and confirmatory testing SOPs, and test follow-up procedures during pilot implementation.

#### Develop expertise-sharing models

Stakeholders proposed ways in which some screening methodologies could be moved out of state laboratories to locations with the necessary expertise to conduct screening or second-tier testing. These approaches would maintain state oversight and encourage resource sharing while alleviating implementation burdens on state laboratories.

One proposed solution was the creation of regional NBS laboratories with expertise and capacity in particular methodologies. This model, in which multiple states would send blood spots to the regional laboratory, was proposed to either span all of NBS or to be used for laboratory methodologies that require technological expertise not available in most states. This model would allow states to specialize in some conditions and associated laboratory methodologies and become a regional laboratory for other states while outsourcing other conditions for which they have insufficient equipment or expertise. This regionalization approach was anticipated to potentially streamline processes while reducing overall costs and burden associated with NBS in each state (Table 5, Quote 5.2.a.).

Another proposed solution was to create or expand collaboration between state NBS programs and universities and academic medical centers. For example, states could outsource screening for certain conditions to university laboratories, and NBS could become more closely aligned with academic medical centers such that states draw from screening, confirmatory testing, interpretation, data analysis and reporting, and follow-up expertise of academic partners rather than bringing new expertise into state laboratories (Table 5, Quote 5.2.b.). These expertise-sharing solutions may increase state-level resources and capacity and support systematic data collection and analysis, especially as screening and confirmation approaches become more complex.

#### Develop a public-private partnership to increase resources and reduce burden on the NBS system

Stakeholders described the creation of a consortium of industry, state and federal government, academic partners, and other stakeholders as a possible facilitator of other proposed solutions (e.g., expansion of pilot studies and expertise-sharing models). They suggested that a public-private partnership could provide scientific leadership, oversight, and funding for NBS implementation, ongoing data collection, and reporting of outcomes (Table 5, Quote 5.3.). For example, private companies involved in therapeutics could fund pilot studies or aspects of screening implementation and in turn be benefited by funding associated with identification of babies who need the treatment they have developed. Private funding could support academic-state partnership models such as regional laboratories or conducting NBS screening and follow-up through academic medical centers, either as the sole funder or supplemented by federal funding streams. Short- and long-term follow-up could also be enhanced by such partnerships, for example by incorporating private genetic counseling organizations into NBS.

#### Other innovative solutions

Such partnerships may provide the resources and expertise needed to pilot even more radical innovations to the system in response to the rapid development of new treatment approaches for rare conditions. One example was the idea of collaborative pilot studies where industry professionals, academic institutions, and states provide screening for a condition with the goal of offering the parents of affected newborns immediate enrollment of their baby in clinical trials. Another example was the idea that conditions would be automatically added to state NBS at the time of new treatment approval by FDA, either as a pilot or as part of the full state panel. Finally, some stakeholders suggested a “conditional RUSP,” where conditions would have a much lower bar for RUSP approval, with the expectation that data would be obtained during implementation and regularly reviewed to determine whether the condition should remain on the RUSP (Table 5, Quote 5.4.),

#### Improve education and public opinion about NBS

Stakeholders suggested opportunities to increase awareness and educate the public about NBS through collaboration of parents/patient advocates, federal and state programs, and other stakeholders. Some recommended reframing rare diseases identified through NBS as a collective public health burden, rather than each new NBS condition presenting a standalone example when it comes to justifying costs or benefits of screening (Table 5, Quote 5.5.a.).

Stakeholders also suggested that promoting the benefits of NBS through a public relations campaign could prove beneficial for advocacy and “making a case” for screening to state legislators (Table 5, Quote 5.5.b.). Such a campaign could incorporate the data generated as part of other solutions proposed by stakeholders, such as data on family perspectives of NBS and results of cost-benefit analyses.

## Discussion

NBS is at a critical inflection point. Although the NBS system has been successful at achieving its goals, obvious challenges and inefficiencies will cause major hurdles and delays when lifesaving therapies become available for numerous disorders that are not yet part of the RUSP. The findings from our multistakeholder panels provide important input to inform efforts to modernize NBS in the United States. Participants identified multifaceted challenges that have been previously reported, particularly vital missing data [6, 27–29], inadequate resources [30–33], and processes that are ill-equipped for rapid or large-scale change [30, 31, 33, 34]. Although many of the challenges to NBS are exacerbated by insufficient funding [28, 30, 32, 33], our results suggest that NBS modernization will require systemwide change that includes, but extends beyond, strategic financial investments to support RUSP approval and screening implementation.

Developing and implementing acceptable and efficacious solutions will require new and strengthened collaborations and capacity-building that cannot easily be achieved through the existing NBS system. It is vital that multiple stakeholders are active in developing solutions, and that those stakeholders remain engaged as solutions are implemented and evaluated [35]. Innovations must work within existing federal and state policies or be addressed through legislative changes. The federal government is unable to mandate state public health practice (with a few notable exceptions), as evidenced by the RUSP being a recommended rather than mandated panel, with states having agency in determining their own screening practices. To that end, participants described innovative ways to leverage strengths, expertise, and resources of stakeholder groups to benefit the overall system.

Participants envisioned both practical and innovative approaches to future NBS. Some of these require only modest change and could be achieved through active collaboration and support of existing NBS state, federal, and academic partners. One example is developing new approaches to regional expertise and expertise sharing. This would allow states to identify their priorities for internal capacity building and share that expertise with others in the region, while relying on reciprocal screening arrangements with other laboratories. The expansion of pilot studies is another approach to develop new collaborations that would result in important data while allowing states an opportunity to implement screening for conditions that are not yet on the RUSP. However, maximizing impact will require addressing multiple limitations, such as the lack of required, standardized collection of natural history and outcomes data that can be used to inform future NBS decision making. It was suggested that more standardized data collection could be facilitated by cross-agency coordination on the federal level.

Other approaches suggested by participants, such as implementing a public-private partnership, have been considered in the NBS context [33] and could dramatically change the way NBS is led, funded, and conducted in the United States if implemented on a larger scale. Such an approach brings exciting opportunities for innovation but also potential conflicts of interest that must be carefully examined and mitigated. NBS stakeholders’ focal point on the infant’s well-being provides a guiding principle against which to deliberate and negotiate on conflicts of interest, in that conflicts that potentially negatively impact the infant and family should be considered differently than those that do not.

The focus on enhancing infant well-being also provides stakeholders with a shared framework to evaluate the potential benefits and harms of other proposed (and yet to be proposed) innovations to NBS. For example, the intriguing concept of linking NBS pilots to the drug development process highlights competing potential benefits and harms to infants and their families. Newborn identification makes trial participation available as an option for more parents of newborns with rare conditions, which may provide hope and a potential for a better outcome for the child. Additionally, conducting NBS pilots to identify children who can be recruited to ongoing clinical trials would very likely speed up the drug development process, since recruitment of infants with rare disorders is time consuming and costly for trial sponsors [25, 36]. This could result in faster access to lifesaving and approved therapies for children around the world. And yet it should not be assumed that trial participation will be acceptable and appealing for all parents, and moreover that all children will meet inclusion criteria or that all families will have the resources (such as parent time, ability to miss work, the ability to travel to sites) necessary to make trial participation possible. Most important is the need to avoid therapeutic misconception (i.e., a failure to appreciate that the purpose of clinical research is to produce generalizable knowledge, regardless of the potential for individual benefit) in the NBS system [37]. Clinical trials do not provide treatments to affected children, but rather test an experimental drug to determine whether it is safe and whether it works.

Another innovative solution was the concept of conditional RUSP approval, whereby conditions could be approved using a less stringent set of criteria but then periodically be reevaluated based on emerging data from state implementation. Such an approach permits the implementation of a “learning system” that could support infant well-being through enhanced access to disease modifying therapies in the presymptomatic or early symptom stage. And yet conditional approval based on a lower threshold of evidence could result in challenges such as unacceptably high false positive or false negative rates. It could lead to increased uncertainty about which infants need treatment, and when (i.e., based on insufficient natural history data and unexpected disorder heterogeneity), potentially exposing infants to unneeded treatment-related risk and parents to anxiety and burden. Finally, it may be challenging for states to remove a condition from the state’s panel once screening for that condition begins, even if emerging evidence leads to the conditional approval being revoked. Stakeholders across the system should be engaged in this type of deliberation to weigh the potential benefits, harms, and limitations of approaches to NBS modernization.

The findings generated by our multistakeholder panels are an important first step in support of system change to pave the way for next generation NBS. While our study was focused on the NBS system in the United States, the findings will have some applicability to NBS in other countries. The themes that emerged do not reflect consensus among stakeholders; rather, we present concepts that emerged from their discourse on the future of NBS in the context of the transformative therapy scenario. It was not feasible to compare themes by stakeholder group because many participants had current or past experiences in more than one stakeholder group. It will be important for future research and engagement efforts to include stakeholders with broad expertise and experiences to develop detailed implementation objectives and procedures for NBS in the United States. Additional efforts should examine the applicability of the findings to non-U.S. countries.

## Supplementary Information

**Additional File 1:** Hypothetical scenario and challenges from the literature

## Data Availability

Because of the nature of this work, the small sample, and assurances of confidentiality provided to participants in the consent form, transcripts of panel discussions cannot be shared. We can provide aggregated, de-identified ranking results from each of the five pre-panel surveys upon request.

## Abbreviations

ACHDNC: Advisory Committee on Heritable Disorders in Newborns and Children
CDC: Centers for Disease Control and Prevention
FDA: U.S. Food and Drug Administration
IND: Investigational New Drug
IRB: Institutional Review Board
NBS: newborn screening
RUSP: Recommended Uniform Screening Panel
SOP: standard operating procedure
