# Supplementary material for: Preparing newborn screening for the future: a collaborative stakeholder engagement exploring challenges and opportunities to modernizing the newborn screening system
Source: BMC Pediatr. 2022 Feb 12;22:90. doi: 10.1186/s12887-021-03035-x (PMC8840788; doi:10.1186/s12887-021-03035-x)
Supplement: Supplementary file 1 — Additional File 1: Hypothetical scenario and challenges from the literature [file 12887_2021_3035_MOESM1_ESM.docx]

**Additional File 1: Hypothetical scenario and challenges from the literature**

**It is 2030 – ten years from today. 30 new transformative gene or cell therapies have been approved by the FDA to treat monogenic, non-oncology rare disorders. Please consider the following assumptions. These assumptions may not reflect future reality but will be useful to frame our upcoming group discussion.**

- Each treats a different genetic disorder.
- Each has a valid screening assay that is not prohibitively costly.
- The therapies are curative or significantly disease modifying if given early in life, but much less or not effective if given later.
- The longer-term risks and duration of efficacy are unknown.
- The cost of the therapies will be completely covered by payers (e.g., insurance, Medicaid).

**Below is a set of factors that could make it challenging to rapidly implement screening for 30 conditions. These challenges are based on a review of the literature on newborn screening (NBS). This may not be a complete list of challenges, and you will have the opportunity to suggest additional challenges during the panel activities.**

1. **Critical data will be missing:** Data needed by the federal advisory committee, states, and other stakeholders will be missing – e.g., natural history, pilot implementation studies, long-term follow-up data. The committee may not be able to approve disorder(s) until evidence is generated and states may not have data needed to plan and implement new conditions. Critical data will take time and substantial resources to collect, especially if conducted under a research protocol.
2. **Yet-to-be determined disorder heterogeneity will complicate clinical decision making:** Although the therapies will be transformative, varying phenotypes and the lack of predictive biomarkers will lead to difficult clinical decisions about who needs to be treated, and for whom treatment should occur in the newborn period.
3. **There will be RUSP review burden:** The burden of reviewing 30 disorders in 10 years will overwhelm the current capability of the federal committee and the expert review group.
4. **There will be implementation burden for state NBS laboratories:** States will need new funding to build disease-specific infrastructure and hire and train new laboratory staff to develop, validate, establish, and adopt new screening procedures.
5. **Major expansion of state bioinformatics capabilities will be needed:** States will need to significantly increase data capacity and develop new expertise to generate, process, and store data and interface with outside data sources.
6. **State-level implementation will take considerable time:** State rules and regulations, combined with funding and capacity limitations mean that it will take many years for most states to implement screening for this many new disorders.
7. **Accessible state follow-up programs will need to be developed and implemented:** Accessible genetic counseling, specialty services, and specialized treatment centers will need to be available for each condition.
8. **There will be uncertainties about long-term benefits and risks of transformative therapies:** This uncertainty will require primary care and specialist clinicians to expand shared decision making and support for families to facilitate informed decisions about treatments.
9. **Educational materials and resources will need to be developed:** These will be required for families, clinicians, and the public. The materials will need to be accessible and updated regularly to reflect emerging findings.
